# Extensive colorectal lymphomatous polyposis complicated by acute intestinal obstruction: a case report

**DOI:** 10.1186/s13256-017-1340-1

**Published:** 2017-07-13

**Authors:** Jaques Waisberg, Amanda do Val Anderi, Pedro Augusto Soffner Cardoso, José Henrique Miranda Borducchi, Demetrius Eduardo Germini, Maria Isete Fares Franco, Cidia Vasconcellos

**Affiliations:** 1 Department of Surgery, Hospital do Servidor Público Estadual de São Paulo, Avenida Ibirapuera 981, 2o andar – Vila Clementino, Sao Paulo, SP 04029-000 Brazil; 2 Department of Pathology, Hospital do Servidor Público Estadual de São Paulo, Sao Paulo, SP 04029-000 Brazil

**Keywords:** Mantle cell lymphoma, Lymphoma, Non-Hodgkin lymphoma, Intestinal obstruction, Intestinal polyposis, Colorectal neoplasms

## Abstract

**Background:**

Multiple lymphomatous polyposis is a rare type of gastrointestinal lymphoma that extensively infiltrates the intestine. Multiple lymphomatous polyposis originates from the mantle zone of the lymphoma follicle and is considered to be a mantle cell lymphoma, which is a relatively aggressive type of B-cell non-Hodgkin’s lymphoma. We report an unusual case of a patient with multiple lymphomatous polyposis with extensive colorectal involvement and acute intestinal obstruction, an atypical complication of this rare disease. On the basis of this case study, the pitfalls in gastrointestinal tract lymphomatous polyposis diagnosis and prognosis, as well as the treatment options, are discussed.

**Case presentation:**

Our patient was a 76-year-old white woman with asthenia, cramps, and swelling in the lower left quadrant of the abdomen, as well as weight loss within the previous 5 months. A colonoscopy revealed polyps in the rectum, sigmoid colon, descending colon, and right and left colic flexures. A biopsy revealed lymphomatous infiltration of the intestinal wall. Because of the large size of the polypoid masses, which narrowed the colonic lumen in multiple locations, the patient developed acute intestinal obstruction and was referred for laparotomy. She underwent a total proctocolectomy with a permanent ileostomy and a left salpingo-oophorectomy. Microscopic examination showed the presence of a multicentric, low-grade, small lymphocytic lymphoma. Immunohistochemical analysis revealed positive immunostaining for CD79a, CD20, and CD45. These results were consistent with the diagnosis of mantle cell lymphoma. Two weeks after surgery and prior to discharge, but before the beginning of chemotherapy, the patient’s general condition worsened as she experienced a severe and progressive respiratory tract infection, advanced respiratory insufficiency, and septic shock, and she ultimately died.

**Conclusions:**

Mantle cell lymphoma develops as a progressive and aggressive disease with widespread polyposis of the gastrointestinal tract. The intensive chemotherapeutic regimens usually result in the regression of macroscopic and microscopic lesions; however, remissions are short in duration, and the median length of patient survival is 3–4 years. Mantle cell lymphoma is a rare disease that should be part of the differential diagnosis of polypoid diseases of the large intestine.

## Background

Although the intestinal diffuse mucosa-associated lymphoid tissue (MALT) found in the mucosa of the digestive system, including the tonsils, Peyer’s patches, and appendix, contains the most lymphocytes of any organ in the immune system following the compartment that comprises the peripheral lymph nodes and spleen, only 10% of all lymphomas occur in the gastrointestinal (GI) tract [1–3]. Non-Hodgkin’s lymphoma (NHL) of the GI tract is the most frequent extranodal lymphoma. Approximately 15–30% of primary extranodal lymphomas occur in the GI tract, and primary GI tract lymphomas account for only 1–10% of all GI malignancies [1–3].

Mantle cell lymphoma, a distinct clinicopathological entity of NHL, is an aggressive B-cell neoplasm that is characterized first by a monotonous proliferation of small- to medium-sized lymphocytes coexpressing CD5, CD20, and cyclin D1 epitopes and second by frequent t(11;14)(q13;q32) chromosomal translocation [4–7]. Mantle cell lymphoma comprises 2.5–7.0% of all NHLs, and the GI tract is involved in approximately 20% of cases [8, 9]. Mantle cell lymphoma is often diagnosed at an advanced stage. The response rate to multiagent chemotherapy is nearly 90%, and the expected 5-year survival rate in selected cases is close to 60% [2, 8–10]. Importantly, mantle cell lymphoma is clinically aggressive and has a shorter median survival period than other types of small B-cell neoplasms, such as the extranodal marginal zone of MALT lymphoma and follicular lymphoma [10–12].

Lymphomatous polyposis of the GI tract is a rare, heterogeneous group of small B-cell lymphomas including mantle cell lymphoma, follicular lymphoma, and MALT lymphoma [3]. Multiple lymphomatous polyposis (MLP) is characterized by the presence of multiple lymphomatous polyps along one or more segments of the digestive tract [2, 9, 10]. MLP is characterized by multiple white, superficially infiltrated nodular or polypoid lesions involving long segments of the GI tract [12]. In most MLP cases, lymphoma cells originate from the mantle zone of the lymphoid follicle, so this disease is actually considered a subtype of mantle cell lymphoma [8, 9], which accounts for 2–9% of primary GI lymphomas [2, 9, 10].

Lymphoma of the colorectal region is mostly of B-cell lineage, as it is in other sites in the GI tract. Cornes [13] coined the disease’s name, *multiple lymphomatous polyposis*, in 1961 to designate numerous polypoid lesions in the GI tract comprising lymphoma with mucosal involvement. Since this first description, only a few cases have been reported [2, 9, 10]. We describe and discuss the clinical and pathological features of a rare case of a patient with mantle cell lymphoma with diffuse involvement of the colon and rectum and complicated by an intestinal obstruction.

## Case presentation

A 76-year-old white woman presented to our hospital with asthenia, cramps, and swelling in the lower left quadrant of the abdomen, as well as weight loss within the previous 5 months. A physical examination revealed pallid mucosa and no palpable superficial lymph nodes. Her Eastern Cooperative Oncology Group/Karnofsky Performance Status score was 1/80. Blood test examinations upon admission revealed anemia (hemoglobin 8.2 g/dl), a hematocrit level of 24.6%, and a white blood cell count of 9300/μl (neutrophils 72%, lymphocytes 20%), as well as proteinemia, albuminemia, carcinoembryonic antigen, blood urea nitrogen, creatinine, lactate dehydrogenase, and human immunodeficiency virus (HIV) serology that were in the normal ranges. Her myelogram result was normal. A colonoscopy was performed because of her complaints of weight loss, fatigue, and anemia and the fact that she was elderly. This procedure revealed polyps in the rectum, sigmoid colon, descending colon, and right and left colic flexures. A rectal biopsy revealed lymphomatous infiltration of the intestinal wall. The pathological diagnosis was of primary colorectal lymphoma. A computed tomography (CT) scan of the patient’s abdomen and pelvis did not reveal extraintestinal involvement, and her liver and spleen were apparently normal. A thoracic CT scan did not show alterations related to lymphoma. A gastroduodenoscopic examination revealed no important findings.

**Fig. 1:**
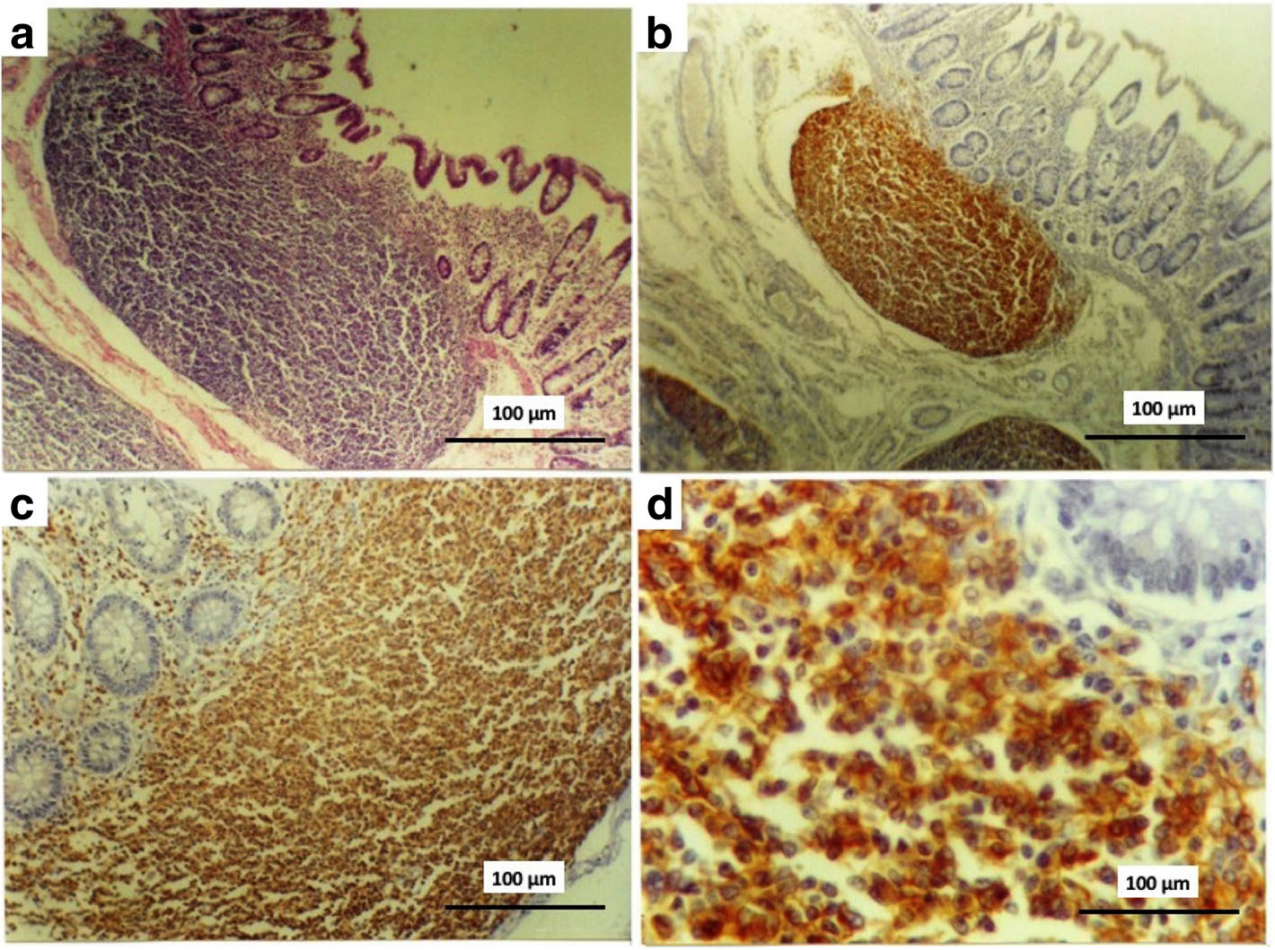
Pathological findings of mantle cell lymphoma. **a** Neoplastic cells involving the small intestine (hematoxylin and eosin counterstain, original magnification ×100). The cell proliferation can be nodular or diffuse, with a mixed nodular pattern. **b** The CD20 antigen was positive in neoplastic cells (immunoperoxidase with hematoxylin counterstain, original magnification ×40). Tumor cells are small- to medium-sized and are composed of a dense, monotonous infiltration of small to intermediate cleaved cells with small, irregularly shaped nuclei and scanty cytoplasm. **c** The CD79a antigen was positive in neoplastic cells (immunoperoxidase with hematoxylin counterstain, original magnification ×200). **d** The CD20 antigen was positive in neoplastic cells (immunoperoxidase with hematoxylin counterstain, original magnification ×400)

Owing to the large size of the polypoid masses, which narrowed the colonic lumen in multiple locations, the patient developed acute intestinal obstruction and was referred for laparotomy. During surgery, a large polypoid lesion was found to be causing an intestinal obstruction in the descending colon, and periaortic lymph nodes with increased dimensions were exposed. A frozen section biopsy of these lymph nodes revealed a well-differentiated small lymphocytic lymphoma. The surgical team decided to remove the colorectal segments involved in lymphomatosis because new episodes of intestinal obstruction could occur and because, in the presence of large intestinal tumors such as these, chemotherapy can lead to intestinal perforation. Thus, the patient was referred for a total proctocolectomy with a permanent ileostomy and a left salpingo-oophorectomy. Macroscopic examination of the surgical specimen revealed colorectal mucosa with polypoid nodules of variable size (1–5 cm in diameter) situated from the ascending colon to the rectum. Microscopic examination revealed the presence of a multicentric, low-grade, small lymphocytic lymphoma originating from the mantle zone of the lymphoid follicle in both the tumor-infiltrated colorectal mucosa and the pericolic/perirectal fat (Fig. 1). Moreover, the lymphoma had also infiltrated the left ovary and fallopian tube as well as all of the 43 dissected mesenteric lymph nodes. Immunohistochemical analysis revealed positive immunostaining for CD79a and CD20 (Fig. 1), as well as CD45, but there was no immunostaining for CD10 or CD23. These results were consistent with a diagnosis of mantle cell lymphoma. The final diagnosis was made 5 months after the onset of symptoms. In this case, the stage of the disease at presentation was assumed as stage II (locally advanced disease). In the postoperative period, the patient received intravenous fluid replacement and antibiotics. Two weeks after surgery and before discharge and the initial chemotherapy, the patient presented with a worsening of her general condition, a respiratory infection caused by pneumonia with fulminant evolution, and septic shock. This status was accompanied by progressive respiratory insufficiency leading to death.

## Discussion

Any part of the GI tract may be involved in mantle cell lymphoma, but diffuse GI involvement is rare [2, 4, 12, 14, 15]. The colon and rectum are usually the most affected segments (approximately 90% of mantle cell lymphoma cases, including this one), followed by the small intestine, stomach, and duodenum [2, 12]. The ileocecal region is frequently the original focus of the mantle cell lymphoma, and it generally remains the primary site of the disease [2, 9]. The macroscopic appearance of mantle cell lymphoma in the GI tract is variable, and it can look like tumor masses, ulcers, mucosal thicknesses, or multiple polypoid lesions, with the latter being characteristic of MLP [2, 9]. The main extradigestive sites affected are the bone marrow, peripheral lymph nodes, Waldeyer’s ring, and liver [2, 9].

Mantle cell lymphoma expresses B-lymphocyte markers (CD20 and CD79a), T-lymphocyte markers (CD5), and cyclin D1. These markers are used in routine immunohistochemistry to characterize the mantle cell lymphoma. However, CD10 is a marker of diffuse, large B-cell lymphoma, and CD23 is a marker for follicular lymphoma; both are usually negative in mantle cell lymphoma. In our patient, immunohistochemical analysis for lymphoma revealed positive immunostaining for CD79a, CD20, and cyclin D1, but there was no immunostaining for CD10 or CD23. A conclusive diagnosis of MLP requires the histological examination of a specimen in a histomorphological, immunohistochemical, and/or immunophenotypic study [6, 7, 16].

Most MLP cases occur in male patients over 50 years of age (median age 60 years) [2, 9, 17]. In general, patients with MLP have no specific clinical manifestations, but the most frequent symptoms are abdominal pain, diarrhea, palpable masses, melena, hematochezia, weight loss, and fatigue [2, 9, 18]. The patient in this study complained of fatigue, diarrhea, abdominal pain, and weight loss, but not of palpable masses or GI bleeding. Less frequently, intestinal obstruction, protein-losing enteropathy, intestinal malabsorption, chylous ascites, and acute abdomen consequent to perforation have also been reported [2, 9, 10, 14, 17]. In the acute intestinal obstruction in cases of MLP, the most frequent manifestation consists of multiple intussusceptions that usually occur in the ileocecal region [14, 18]. The mean interval between the onset of symptoms and the diagnosis is 3 months (range 1–9 months), which was the interval for our patient [2, 9, 10, 17]. By the time of diagnosis, more than half of the patients have bone marrow infiltration and involvement of the liver, spleen, and peripheral and mesenteric lymph nodes [2, 9, 12]. Our patient showed a significant infiltration of the mesenteric lymph nodes but had clean bone marrow, liver, spleen, and peripheral lymph nodes.

In general, small or nodular polypoid tumors and sessile tumors of different sizes are found throughout the GI tract during upper GI endoscopy, enteroscopy, or colonoscopy examinations that are performed to confirm the locations of the polyps and to collect tissue biopsies [2, 9, 10, 12]. Our patient presented with many large polyps, some with a diameter up to 5 cm, which are capable of occluding the intestinal lumen.

Most MLPs originate from mantle cell lymphoma, but others originate from follicular lymphoma or MALT lymphoma. Other entities need to be included in the differential diagnosis, particularly regarding chronic lymphoid leukemia; peripheral T-cell lymphoma; subtypes of lymphocytic lymphoma such as diffuse large B-cell lymphoma; and immune proliferative small intestinal disease, a subtype of MALT lymphoma [9, 10, 17, 19]. It is also judicious to differentiate MLP from colonic polyposis-like adenomatous polyps, familial adenomatous polyposis, and variant syndromes such as Turcot, Gardner, Peutz-Jeghers, and Cronkhite-Canada syndromes [9, 15].

Current multiagent chemotherapy and arrangements of monoclonal antibodies have led to important progress in mantle cell lymphoma response rates. The overall response rates fluctuate from 80% to 95%, and complete response rates of 30–50% are often reached [8, 9, 20–27].

For patients in good condition who are younger than 65 years of age, the treatment of choice is an intensive immunochemotherapy combining rituximab with cyclophosphamide, doxorubicin, vincristine, and prednisone (R-CHOP) [20–22]. A therapy with single-agent rituximab, a chimeric monoclonal antibody that binds specifically to the CD20 antigen, produced response rates of approximately 30%; when combined with an anthracycline-containing regimen, the response rate can increase to more than 90% [22–24]. Standard immunochemotherapy with R-CHOP reduces lesions, and the initial response to treatment reaches 94% [20–22]. Nevertheless, the resulting remissions are of short duration, and the thorough response rate is only 31% [22, 23].

Other alternative treatments include a regimen of dexamethasone, high-dose cytarabine, and cisplatin or one of hyperfractionated cyclophosphamide, vincristine, doxorubicin, dexamethasone, methotrexate, and high-dose cytarabine (hyper-CVAD). After disease control, therapy can be followed by autologous peripheral blood stem cell transplant. The initial therapeutic response rate of the hyper-CVAD regimen is over 90% [24].

However, the general poor outcome of MLP has led to the use of more aggressive therapies. Myeloablative therapies with stem cell support have significantly improved outcomes. If the patient is in good physical condition and is younger than 65 years old, intensive frontline immunochemotherapy with a multidrug regimen, followed by an autologous stem cell transplant, should be considered [2, 24, 28]. However, nonintensive strategies are reserved for older patients over 65 years of age or those who have important comorbidities. Intensive immunochemotherapy, both with and without stem cell support, has been used effectively to extend progression-free survival to 5 years or more [2, 22, 28–30]. In relapsed or refractory cases, hyper-CVAD plus rituximab can be an alternative therapeutic scheme [22–24].

For older patients, those who cannot receive a peripheral blood stem cell autograft, or those with an anthracycline contraindication, purine nucleoside analogues associated with rituximab are a plausible option [8, 23, 25]. Surgery may be appropriate and/or necessary for patients with complications such as recurrent GI bleeding and perforation or obstruction of the intestine [14, 18], as in our patient.

Mantle cell lymphoma has one of the poorest prognoses of all NHL subtypes, with a median survival time of less than 3 years [2, 9, 10, 25, 26] and frequent relapses [3, 8, 27]. The poor prognostic factors include unsatisfactory general clinical condition, involvement of multiple extranodal sites, advanced age (older than 70 years), elevated lactate dehydrogenase levels, and bone marrow infiltration [2, 9, 10, 17]. Several morphological variants can be recognized in peculiar aggressive variants, either blastoid or pleomorphic and adverse prognosis [2]. To identify subsets and classify patients with mantle cell lymphoma according to their prognoses, several researchers have attempted to establish a prognostic index. Ki-67 positivity, which represents cell proliferation, has been reported as a predictor. The number of Ki-67-positive cells lies between 5% and 50% of the cells [2]. In cases of high positivity for the Ki-67 cell proliferation index, high mitotic rates are associated with adverse prognoses [23, 26, 27]. The expected 5-year survival rate in selected cases is close to 60%, which is lower than that for other GI B-cell NHLs [2, 9, 10, 20, 25].

## Conclusions

Mantle cell lymphoma develops as a progressive disease with a relatively aggressive clinical evolution and a high response rate to multiagent chemotherapy, but the outcomes may be poor in some cases, particularly in older patients. In the GI tract, so-called multiple lymphomatous polyposis is the counterpart of peripheral mantle cell lymphoma. Mantle cell lymphoma must be considered in patients with widespread polyposis of the GI tract. Prognosis has been significantly improved since, in younger patients, intensive frontline immunochemotherapy with autologous stem cell transplantation was proposed.

## Abbreviations

CT: Computed tomography
GI: Gastrointestinal
Hyper-CVAD: Hyperfractionated cyclophosphamide, vincristine, doxorubicin, dexamethasone, methotrexate, and high-dose cytarabine
MALT: Mucosa-associated lymphoid tissue
MLP: Multiple lymphomatous polyposis
NHL: Non-Hodgkin’s lymphoma
R-CHOP: Combination of rituximab with cyclophosphamide, doxorubicin, vincristine, and prednisone
